# Knowledge, attitudes, and practices of patients with recurrent pregnancy loss toward pregnancy loss

**DOI:** 10.3389/fpubh.2023.1308842

**Published:** 2024-01-11

**Authors:** Fangxiang Mu, Tianyu He, Kexin Wang, Fang Wang

**Affiliations:** Department of Reproductive Medicine, Lanzhou University Second Hospital, Lanzhou, China

**Keywords:** knowledge, attitude, practices, recurrent pregnancy loss, cross-sectional study

## Abstract

**Objective:**

Self-management is crucial in managing recurrent pregnancy loss (RPL). This study explored the knowledge, attitudes, and practices (KAP) of patients with RPL toward RPL.

**Methods:**

This cross-sectional study was conducted among patients with RPL between January 2023 and June 2023 at the Second Hospital of Lanzhou University. Participants’ demographic characteristics and KAP were determined using a self-designed questionnaire (Cronbach’s α = 0.818). Structural equation modeling (SEM) was used to observe the correlations among KAP and different factors.

**Results:**

This study analyzed 497 valid questionnaires. The mean knowledge, attitude, and practice scores were 11.59 ± 4.30 (possible range: 0–20, 57.95%), 44.17 ± 3.18 (possible range: 13–65, 67.95%), and 32.39 ± 5.22 (possible range: 8–40, 80.98%), indicating poor knowledge, moderate attitude, and proactive practice. Age was non-linearly associated with the KAP dimensions, with a positive impact of age on KAP among those aged <32 years old. Knowledge was directly influenced by education (β = 1.49, *p* < 0.001) and income (β = 1.08, *p* < 0.001). The attitude was directly influenced by knowledge (β = 0.25, *p* < 0.001) and indirectly influenced by education (β = 0.37, *p* = 0.001) and income (β = 0.27, *p* < 0.001). Practice was directly influenced by knowledge (β = 0.26, *p* < 0.001), attitude (β = 0.28, *p* < 0.001), and income (β = 0.68, *p* = 0.012), and indirectly influenced by knowledge (β = 0.07, *p* = 0.001), education (β = 0.59, *p* = 0.001), and income (β = 0.42, *p* < 0.001).

**Conclusion:**

Women with RPL in Lanzhou show poor knowledge, moderate attitude, and proactive practice toward RPL. This study identified specific KAP items that would require improvements. The study also identified categories of patients who would need more attention.

## 1 Introduction

Recurrent pregnancy loss (RPL) is defined as ≥2–3 consecutive losses of clinical pregnancy documented by ultrasound or histopathologic exam (1–3). Primary RPL refers to no prior live births or pregnancies beyond 20–24 weeks gestation (4). Secondary RPL refers to ≥1 previous live birth or pregnancy beyond 20–24 weeks gestation (4). Clinically recognized pregnancy loss is reported in about 15–25% of pregnancies (1), and the reported prevalence of RPL is 1–4% of all patients who achieve pregnancy (4). A large-scale study in China (299,580 women) revealed a rate of RPL of 1.92% (5). In Lanzhou (China), the natural miscarriage rate is 14.64% (6). The cause of RPL is reported to be idiopathic in 50–75% of cases, although it may be associated with increasing parental age (1–4). Antiphospholipid antibodies are reported in 8–42% of patients with RPL, embryonic chromosomal abnormalities are reported in 30–57%, and congenital uterine malformation is reported in 1.8–37.6% (1, 2, 4). The general management considerations for all couples/patients with RPL include psychological support and counseling, dietary and lifestyle changes (3), and referral to a specialist clinic (2). Genetic counseling is recommended, while *in vitro* fertilization with preimplantation genetic testing is not recommended to treat RPL because it has not been shown to improve live birth rates (2–4). Patients with identified causes of RPL (e.g., thrombophilia, metabolic or hormonal abnormalities, and uterine abnormalities) should first have their condition managed (3).

Hence, the management of RPL requires the active participation of the women. Indeed, women with RPL should seek psychological and genetic counseling. The management of RPL also involves changes in lifestyle habits, including taking multivitamins and vitamin D supplements, stopping smoking, attaining and maintaining a normal range body mass index (BMI), and limiting alcohol consumption (2). Women should also advise their spouses to make the same changes in lifestyle habits (3). Therefore, the management of RPL actively involves self-management. Still, a basis of self-management is a proper knowledge of the disease and its management (7). The results of knowledge, attitude, and practice (KAP) can be used to design educational interventions to improve the management of a specific condition (8, 9). Knowledge is the basic information and data required as the basis to have motivation toward a given subject and practice. Attitude is the motivation toward a given subject and is the force driving practice (8, 9). Practice is the actions taken to perform and apply the subject. The KAP theory entails that practices can be changed by modulating attitudes through knowledge.

In Saudi Arabia, about 50% of women had knowledge of RPL, but the KAP was generally poor (10), as supported by another study from Egypt (11). A previous study in Qatar revealed numerous misconceptions about RPL (12). Healthcare providers are often a primary source of medical information for patients, and such professionals often display poor knowledge of miscarriage (13). Still, KAP is highly dependent upon the culture, society, economic status, and customs and can vary among countries. No KAP data regarding RPL are available from China.

China participates in the Millennium Development Goals (MDGs). As one of the biggest developing countries, China achieved great progress in improving maternal health by achieving the MDG 5.A (i.e., reducing maternal mortality by 75% between 1990 and 2015) and MDG 5.B (i.e., universal access to reproductive health services) (14, 15). China also applies the Sustainable Development Goals (SDGs) 3, which entails that a country makes efforts to achieve universal health coverage with equal access to affordable, accountable, and appropriate health services of certified quality (16, 17). China offers life-round preventive and curative health services to women, with universal access to reproductive health services (14). Still, the reproductive health workforce has been shown to be unequally distributed in the country (15), and efforts are being taken to remedy the situation. The present study was performed in Lanzhou, the capital of Gansu Province. The per-capita gross domestic product ranks 134 among 659 Chinese cities.

The China Consensus on the Diagnosis and Treatment of Recurrent Abortion (2022) (18) emphasizes that post-pregnancy psychological factors, lifestyle, and post-pregnancy monitoring and management should not be overlooked and should be inquired during the miscarriage workup. The exposure to adverse lifestyles and environmental factors should be documented. Psychological assessments and counseling should be provided. Unhealthy lifestyle habits should be corrected. The patients should be closely monitored in early pregnancy with regular checkups and examinations.

Therefore, this study aimed to examine the KAP of patients with RPL toward RPL in Lanzhou. In the context of the present study, knowledge is the knowledge about what recurrent pregnancy loss is, the possible causes, and the possible management methods. Attitude represents what the women think about multiple biochemical pregnancies, the management of RPL, the biological meaning of RPL, the importance of identifying the causes of RPL, the women’s worries about themselves and their spouses, and the women’s trust in physicians. Practice is what the women are actually doing regarding RPL, i.e., if they participate in training, what they do if an abortion is inevitable, and their life behaviors.

## 2 Methods

### 2.1 Study design and participants

This cross-sectional study was conducted between January 2023 and June 2023 at the Second Hospital of Lanzhou University among patients with RPL. The study was approved by the ethics committee of the Second Hospital of Lanzhou University. All participants signed the informed consent form before completing the questionnaire.

The inclusion criteria were (1) history of at least two spontaneous abortions (manifestations of biochemical pregnancy, empty gestational sac, gradual cessation of embryonic development, embryonic or fetal death, and expulsion of the embryo and its appendages; this study was about women with RPL), (2) desire to have a child (the women were seeking medical help for RPL because they were still wanting a child), and (3) ability to use the Chinese language to read and communicate (to be able to complete the questionnaire). The exclusion criteria were (1) <18 years of age (this study only included adults), (2) severe cognitive impairment or previous history of dementia or psychosis (such factors could influence the patient’s grasp of reality or understanding), or (3) unwilling to sign the informed consent form (mandatory for all prospective human studies).

### 2.2 Questionnaire

A self-designed questionnaire with four dimensions was developed based on the *Expert Consensus on the diagnosis and treatment of recurrent abortion (2022)* (18) and was modified according to the comments from two experts in the field of obstetrics and gynecology and one expert in the field of psychology, leading to the deletion of some similar or repetitive questions and the adjustment and refinement of some questions that were not clearly formulated. A small-scale pilot study (50 samples) was conducted. Cronbach’s α was 0.818, indicating a relatively high level of internal consistency.

The final questionnaire consisted of (1) demographic characteristics (age, residence, education, work status, monthly income, BMI, fertility status, live births, and previous abortions), (2) knowledge dimension (for each of the 10 questions in this category, participants were assigned scores based on their responses, with a score of 2 for very well-known, 1 for having heard of it, or 0 for unclear), (3) attitudes dimension [each of the 13 questions in this category was scored on a five-point Likert scale, ranging from very positive (5 points) to very negative (1 point)], and (4) practices dimension [each of the eight questions in this category was also scored on a five-point Likert scale ranging from always (5 points) to never (1 point)]. The total score for each dimension was the sum of the scores of all items within a given dimension, and the mean score for each dimension was calculated using unweighted arithmetic means. Higher scores for each dimension indicated adequate knowledge, more positive attitudes, and more proactive practices. The participants’ overall knowledge, attitude, and practice scores were categorized using a modified Bloom’s criteria cutoff point: 80–100% were considered good knowledge, positive attitude, and appropriate practice, respectively; 60–79% was considered moderate; <60% was considered poor knowledge, negative attitude, and inappropriate practice, respectively (19).

### 2.3 Questionnaire administration

An online questionnaire was constructed using the WeChat-based Questionnaire Star applet. A QR code was generated to collect data through WeChat. The participants scanned the QR code sent via WeChat to log in and complete the questionnaire. The questionnaire’s homepage served as the informed consent for this study. Before distributing the questionnaire, researchers explained the purpose, significance, content, and survey instructions. Researchers were present throughout the survey process and provided on-site supervision. Any questions that research subjects found unclear or did not understand during the survey process were promptly explained by the researchers.

### 2.4 Quality control

In order to ensure the quality and integrity of the questionnaire results, all items were mandatory. The research team members examined the completeness, internal coherence, and reasonableness of all questionnaires. A given IP address could be used only once to submit a questionnaire. Two data management personnel reviewed the data from the previous day in the Questionnaire Star backend system at 9:00 AM daily. Clearly erroneous or illogical data entries were recorded, and feedback was provided to the clinical researchers. Researchers then contacted patients to verify the accuracy of the data. If data errors were identified, data management personnel added comments in the Questionnaire Star backend system to help data cleaning personnel identify data errors.

### 2.5 Statistical analysis

The continuous variables were described using means ± standard deviations (SD) and analyzed using Student’s *t*-test or ANOVA. The categorical variables were described using *n* (%) and analyzed using the chi-square test. Pearson correlation analysis was used to assess the correlations between knowledge, attitude, and practice scores. Restricted cubic splines (RCS) were used to examine the non-linear relationship between age and KAP, with variables included based on logistic univariable analyses with *p* < 0.05. Structural equation modeling (SEM) is a multivariate statistical analysis technique used to analyze structural relationships among the included variables. This technique combines factor analysis and multiple regression analysis, and it is used to analyze the structural relationship between measured variables and latent constructs (20, 21). In the present study, SEM was used to observe the correlations among KAP and different factors. The working hypotheses for the SEM were (1) knowledge directly influences attitude, (2) attitude directly influences practice, (3) knowledge directly and indirectly influences practice, and (4) demographic factors like income, education, and body mass index will directly and indirectly influence KAP dimensions. The analyses were performed using R 4.3.1 (for RCS) and Stata 17.0 (Stata Corporation, College Station, TX, United States; for all other analyses). Two-sided *p*-values <0.05 were considered statistically significant.

## 3 Results

### 3.1 Characteristics of the participants

A total of 500 questionnaires were collected, and 497 (99.40%) were valid questionnaires (one participant completed the questionnaire within 4 s, which is impossible when reading the questions, and two participants reported impossible BMI data that could not be verified). The participants were 32.28 ± 4.28 years old, had a BMI of 22.46 ± 3.03 kg/m^2^, 81.89% were residing in urban areas, and most had a junior college or undergraduate education (71.03%), were employed (62.98%), had a monthly income of 5,000–15,000 (48.49%), were preparing for pregnancy (50.30%), had no previous live births (73.44%), and had two previous spontaneous abortions (51.91%; Table 1).

**Table 1:** Baseline characteristics and KAP scores.

	*n* (%)	Knowledge score		Attitudes score		Practices score	
		Mean ± SD	*p*	Mean ± SD	*p*	Mean ± SD	*p*
Total		11.59 ± 4.30		44.17 ± 3.18		32.39 ± 5.22	
Age (years)	32.28 ± 4.28	-		-		-	
Residence			<0.001		0.037		0.025
Urban	407 (81.89)	12.00 ± 4.24		44.31 ± 3.24		32.64 ± 5.25	
Non-Urban	90 (18.11)	9.74 ± 4.12		43.53 ± 2.81		31.28 ± 4.96	
Education			<0.001		<0.001		<0.001
High school and below	106 (21.33)	9.35 ± 4.38		43.17 ± 2.60		29.88 ± 5.71	
Junior college/undergraduate	353 (71.03)	12.07 ± 4.06		44.36 ± 3.28		32.99 ± 4.95	
Postgraduate and above	38 (7.65)	13.39 ± 4.10		45.16 ± 3.09		33.92 ± 3.96	
Work status			0.002		0.025		0.003
Employed	313 (62.98)	12.17 ± 4.33		44.46 ± 3.19		32.93 ± 5.09	
Unemployed	75 (15.09)	10.63 ± 3.78		43.71 ± 2.87		30.56 ± 5.85	
Self-employed	34 (6.84)	11.00 ± 4.30		43.53 ± 2.50		31.32 ± 5.57	
Housewife	53 (10.66)	10.08 ± 4.67		43.23 ± 3.69		31.94 ± 4.58	
Other	22 (4.43)	11.14 ± 3.27		44.77 ± 3.13		33.73 ± 4.20	
Income, 10, 000 yuan			<0.001		<0.001		<0.001
<5	124 (24.95)	9.80 ± 4.66		43.23 ± 2.58		30.73 ± 5.59	
5–15	241 (48.49)	11.60 ± 4.03		44.19 ± 3.33		32.47 ± 5.06	
16–30	98 (19.72)	12.93 ± 3.76		45.14 ± 3.09		33.76 ± 4.71	
>30	34 (6.84)	14.15 ± 3.72		44.65 ± 3.44		33.97 ± 4.82	
BMI (kg/m^2^)	22.46 ± 3.03						
Fertility status			0.013		0.005		0.011
Pregnancy preparation	250 (50.30)	11.22 ± 4.45		43.70 ± 3.14		31.72 ± 5.10	
Early pregnancy	125 (25.15)	11.42 ± 4.15		44.30 ± 3.30		32.38 ± 5.48	
Mid pregnancy	52 (10.46)	12.25 ± 3.77		44.77 ± 2.95		33.79 ± 4.87	
Late pregnancy	35 (7.04)	11.66 ± 3.77		44.89 ± 3.58		34.03 ± 4.76	
Postpartum	35 (7.04)	13.80 ± 3.87		45.40 ± 2.30		33.54 ± 5.44	
Times of previous live births			0.146		0.372		0.785
0	365 (73.44)	11.76 ± 4.18		44.09 ± 3.19		32.43 ± 5.03	
≥1	132 (26.56)	11.12 ± 4.61		44.38 ± 3.14		32.29 ± 5.75	
Times of previous abortion			0.193		0.934		0.180
2	258 (51.91)	11.25 ± 4.18		44.12 ± 3.14		32.31 ± 5.14	
3	138 (27.77)	11.99 ± 4.37		44.20 ± 3.32		32.53 ± 5.04	
≥4	101 (20.32)	11.89 ± 4.48		44.25 ± 3.11		32.44 ± 5.69	

### 3.2 Knowledge

The mean knowledge score was 11.59 ± 4.30 (possible range: 0–20, 57.95%), indicating poor knowledge. Higher knowledge scores were observed in urban residents (*p* < 0.001), higher education (*p* < 0.001), employment (*p* = 0.002), higher income (*p* < 0.001), and postpartum (*p* = 0.013) (Table 1). The items with the lowest knowledge score were K7 (*“If patients with recurrent abortion are diagnosed with hypothyroidism, they need to take levothyroxine.”*) and K9 (*“Currently, there is no evidence to suggest that vitamin D can reduce the risk of miscarriage, but regular intake of vitamin D supplements may reduce the risk of pregnancy complications”*), while the item with the highest score was K5 (*“5. Although no single treatment can completely prevent recurrent miscarriages, maintaining a healthy lifestyle is still a wise choice”*) (Supplementary Table S1).

### 3.3 Attitude

The mean attitude score was 44.17 ± 3.18 (possible range: 13–65, 67.95%), indicating moderate attitudes. Higher attitude scores were observed in urban residents (*p* = 0.037), higher education (*p* < 0.001), employment (*p* = 0.025), higher income (*p* < 0.001), and postpartum (*p* = 0.005) (Table 1). The item with the lowest attitude score was A7 (*“I frequently worry about the possibility of recurrent miscarriage, leading to feelings of nervousness and anxiety.”*), while the item with the highest score was A8 (*“Family care and support are very important to me.”*) (Supplementary Table S2).

### 3.4 Practice

The mean practice score was 32.39 ± 5.22 (possible range: 8–40, 80.98%), indicating proactive practice. Higher practice scores were observed in urban residents (*p* = 0.025), higher education (*p* < 0.001), employment (*p* = 0.003), higher income (*p* < 0.001), and late pregnancy (*p* = 0.011) (Table 1). The item with the lowest practice score was P3.4 (“Engage in moderate exercise, avoiding obesity.”), while the item with the highest score was P3.2 (“Abstain from alcohol.”) (Supplementary Table S3).

### 3.5 Correlations

Pearson correlation analysis showed the knowledge scores were correlated to the attitude (*r* = 0.368, *p* < 0.001) and practice (*r* = 0.310, *p* < 0.001) scores, while the attitude scores were correlated to the practice scores (*r* = 0.271, *p* < 0.001) (Table 2).

**Table 2:** Pearson correlation analysis.

	Knowledge	Attitude	Practice
Knowledge	1		
Attitude	0.368 (*p* < 0.001)	1	
Practice	0.310 (*p* < 0.001)	0.271 (*p* < 0.001)	1

### 3.6 Restricted spline regression

**Figure 1:**
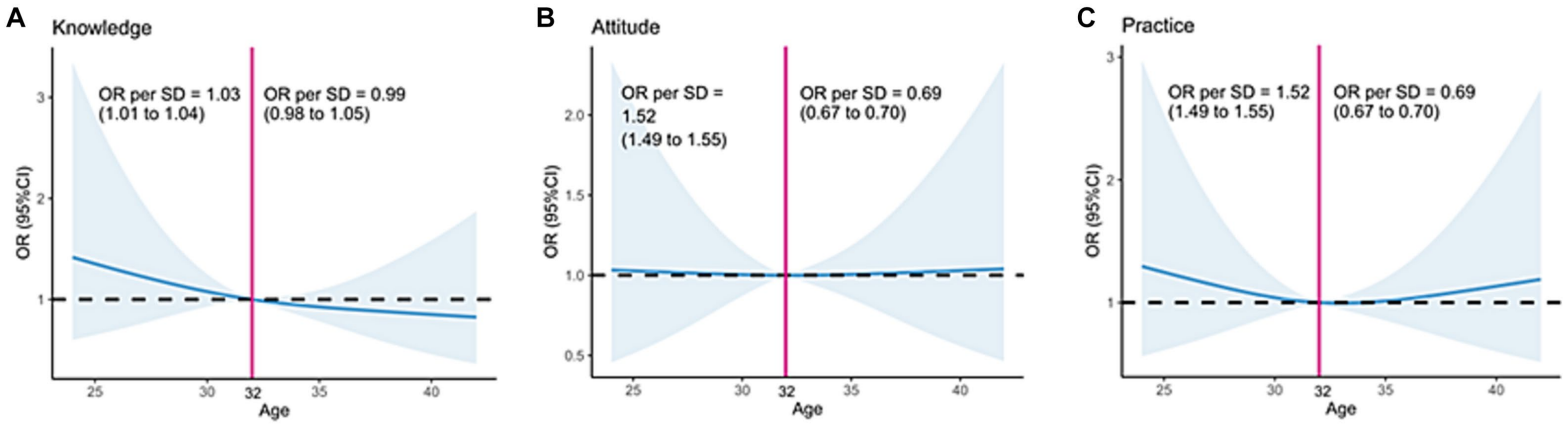
Impact of age on **(A)** knowledge, **(B)** attitudes, and **(C)** practice toward recurrent abortions.

As shown in Figure 1 and Table 3, age was positively associated with knowledge when aged <32 years old (OR = 1.03, 95%CI: 1.01–1.04), but not after 32 (OR = 0.99, 95%CI: 0.98–1.05). Age was positively associated with attitude when aged younger than 32 years (OR = 1.52, 95%CI: 1.49–1.55) but was negatively associated with attitude among participants aged >32 years old (OR = 0.69, 95%CI: 0.67–0.70). Similarly, age was positively associated with the practice when aged <32 years scores (OR = 1.52, 95%CI: 1.49–1.55) but negatively associated after 32 (OR = 0.69, 95%CI: 0.67–0.70).

**Table 3:** Restricted spline regression (RCS).

	*n* (%)
Knowledge	
[0, 14]	364 (73.24)
(14, 20]	133 (26.76)
Attitude	
[13, 45.50]	338 (68.01)
(45.50, 65]	159 (31.99)
Practice	
[8, 28]	103 (20.72)
(28, 40]	394 (79.28)

The moderator variables in knowledge were location, education, job status, income, and pregnancy status. The moderator variables in attitude were education, income, body mass index, and pregnancy status. The moderator variables in practice were education and income.

### 3.7 Structural equation modeling

**Figure 2:**
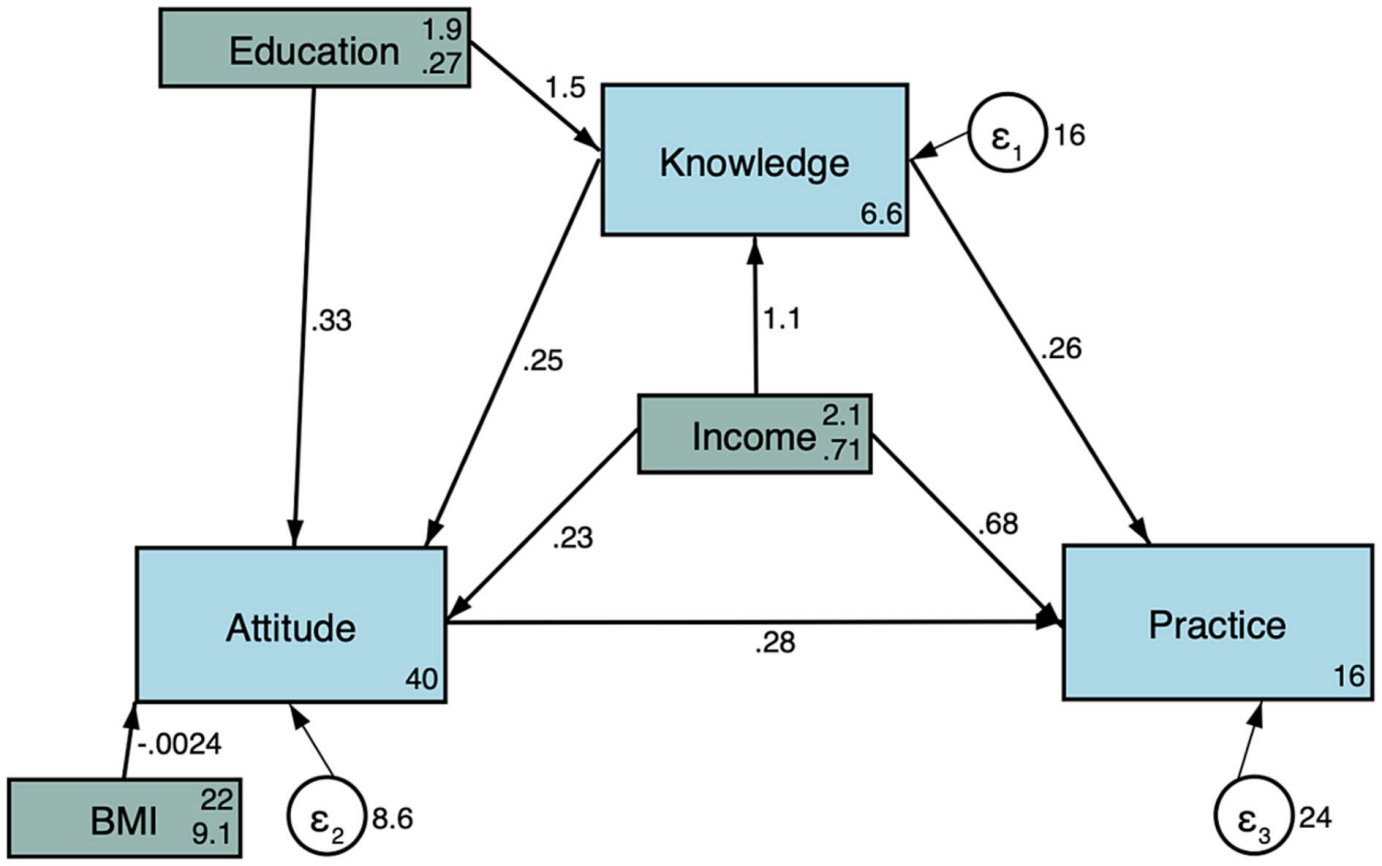
Structural equation modeling.

As shown in Figure 2 and Table 4, knowledge was directly influenced by knowledge (β = 1.49, *p* < 0.001) and income (β = 1.08, *p* < 0.001). The attitude was directly influenced by knowledge (β = 0.25, *p* < 0.001) and indirectly influenced by education (β = 0.37, *p* = 0.001) and income (β = 0.27, *p* < 0.001). Practice was directly influenced by knowledge (β = 0.26, *p* < 0.001), attitude (β = 0.28, *p* < 0.001), and income (β = 0.68, *p* = 0.012), and indirectly influenced by knowledge (β = 0.07, *p* = 0.001), education (β = 0.59, *p* = 0.001), and income (β = 0.42, *p* < 0.001).

**Table 4:** Structural equation modeling.

Model paths	Direct effect		Indirect effect	
	β (95% CI)	*p*	β (95% CI)	*p*
Education → Knowledge	1.49 (0.72, 2.26)	<0.001	-	-
Income → Knowledge	1.08 (0.61, 1.55)	<0.001	-	-
				
Knowledge → Attitude	0.25 (0.18, 0.31)	<0.001	-	-
Education → Attitude	0.33 (−0.23, 0.90)	0.246	0.37 (0.16, 0.58)	0.001
Income → Attitude	0.23 (−0.12, 0.58)	0.195	0.27 (0.13, 0.40)	<0.001
BMI → Attitude	−0.002 (−0.09, 0.08)	0.957	-	-
				
Knowledge → Practice	0.26 (0.15, 0.37)	<0.001	0.07 (0.03, 0.11)	0.001
Attitude → Practice	0.28 (0.14, 0.43)	<0.001	-	-
Education → Practice	-	-	0.59 (0.25, 0.92)	0.001
Income → Practice	0.68 (0.15, 1.21)	0.012	0.42 (0.20, 0.64)	<0.001
BMI → Practice	-	-	−0.001 (−0.02, 0.02)	0.957

Structural equation modeling is a multivariate statistical analysis technique used to analyze structural relationships among the included variables. This technique combines factor analysis and multiple regression analysis, and it is used to analyze the structural relationship between measured variables and latent constructs.

## 4 Discussion

This study suggested that women with RPL in Lanzhou show poor knowledge, moderate attitude, and proactive practice toward RPL. This study identified specific KAP items that would require improvements. The study also identified categories of patients who would need more attention.

Few data regarding the KAP toward RPL are available in the literature. In Saudi Arabia, Tyagi et al. (10) reported that about 50% of the women had some knowledge of RPL and that 60% had positive attitudes, but the study identified several points to be improved, especially regarding the roles of lifting heavy objects, previous contraception methods, and sexually transmitted diseases. Practice regarding medical help also had to be improved. In Egypt, Salama et al. (11) showed that >50% of the women had poor knowledge, but about 67% had positive attitudes. In the present study, the knowledge was poor, but attitudes were moderate, and practice was proactive. Hence, these results suggest that many women followed the physicians’ recommendations without understanding why they performed specific actions to improve their chances of pregnancy.

Age is a major factor affecting fertility, and women are aware that advancing age toward inexorable menopause decreases their chances of having a baby (22, 23). Accordingly, the present study showed that in women younger than 32, age was positively associated with knowledge, attitude, and practice, while after 32, age was negatively associated with attitude and practice, suggesting that with advancing age, the women with RPL are losing confidence in eventually achieving a pregnancy and pay less attention to the self-management to increase the likelihood of pregnancy. On the other hand, although Salama et al. (11) did not analyze age and attitude using non-linear regression, they showed that the frequency of negative attitudes was much more frequent in younger women. It could be due to social differences between Egypt and China and the social, spouse, and peer pressure to achieve a pregnancy. Bailey et al. (24) also showed that women hoped for a pregnancy but expected the worst, especially with advancing age. Tavoli et al. (25) also showed that women generally gained more negative attitudes with the number of recurrent abortions, which was not observed in the present study, but the number of abortions and age are inevitably covariates, and the statistical significance could be lost.

The present study showed that the women with higher education and income had better KAP toward RPL. It is generally supported by the fact that health literacy is directly related to socioeconomic status (26). Accordingly, and supporting the poor knowledge observed in the present study, Munakampe et al. (27) reported poor knowledge of reproductive health in low- and middle-income countries. On the other hand, Campillo et al. (28) reported no association between education and the risk factors for spontaneous abortion, but their study only enrolled university students.

According to the KAP theory, knowledge is the basis for practice, while attitude is the force driving practice (8, 9). In the present study, the Pearson and SEM analyses showed that knowledge positively influenced attitude and practice, and attitude positively influenced practice. Hence, improving knowledge should directly impact attitude and practice. Especially, the definition of RPL, the etiology of RPL, and the management of comorbidities that can increase the risk of RPL should be properly taught to the general population. Such misconceptions were also previously observed in Saudi Arabia (10) and Qatar (12). Still, a study showed that Flemish midwives had a poor knowledge of RPL (13). Since healthcare providers are a major source of medical information for many people, such providers working with pregnant women should be properly trained on RPL.

This study has limitations. The study was conducted at a single center, limiting the sample size and the generalizability of the study. Indeed, healthcare literacy and KAP are often associated with socioeconomic status (26), which varies widely among geographical areas in China (29, 30). Although it was a prospective study, the questionnaire was self-designed by the investigators according to the local practices and policies, limiting generalizability. The study was cross-sectional, preventing any analysis of causality. A SEM analysis was performed to provide some clues about causality, but the causality was statistically inferred rather than observed, and such results must be interpreted with caution. In addition, the results represent the KAP at a precise point in time but could be used as a historical baseline to examine the impact of future education interventions. All KAP studies are at risk of social desirability bias, in which the participants can be tempted to answer what they know they should do instead of what they are doing (31, 32). Considering that the practice scores were high, that bias is a possibility. Multicenter studies and pre/post-intervention studies should be performed to confirm the results.

In conclusion, women with RPL in Lanzhou show poor knowledge, moderate attitude, and proactive practice toward RPL, and patients’ age displays a non-linear relationship with KAP. This study identified specific KAP items that would require improvements. The study also identified categories of patients who would need more attention.

## Data availability statement

The original contributions presented in the study are included in the article/Supplementary material, further inquiries can be directed to the corresponding author.

## Ethics statement

The studies involving humans were approved by the ethics committee of the Second Hospital of Lanzhou University. The studies were conducted in accordance with the local legislation and institutional requirements. The participants provided their written informed consent to participate in this study.

## Author contributions

FM: Conceptualization, Writing – original draft. TH: Data curation, Writing – original draft. KW: Data curation, Writing – original draft. FW: Conceptualization, Data curation, Writing – review & editing.
