# Supplementary material for: Knowledge, attitudes, and practices of patients with recurrent pregnancy loss toward pregnancy loss
Source: Front Public Health. 2024 Jan 11;11:1308842. doi: 10.3389/fpubh.2023.1308842 (PMC10808478; doi:10.3389/fpubh.2023.1308842)
Supplement: Supplementary file 1 [file Table_1.DOCX]

**Supplementary Table S1.** Knowledge of the participants

| **Statement** | **Very well known** | **Heard of** | **Unclear** |
| --- | --- | --- | --- |
| 1. In our country, abortion includes biochemical pregnancies but does not include ectopic and molar pregnancies. | 100 (20.12) | 297 (59.76) | 100 (20.12) |
| 1. The etiology of recurrent abortion is highly complex, and despite many associated factors, many women still cannot identify specific causes. | 150 (30.18) | 302 (60.76) | 45 (9.05) |
| 1. Chromosomal abnormalities in embryos are recognized as a common cause of natural abortion or recurrent abortion. | 161 (32.39) | 275 (55.33) | 61 (12.27) |
| 1. Autoimmune abnormalities are closely related to recurrent abortion, with common diseases including antiphospholipid syndrome, systemic lupus erythematosus, undifferentiated connective tissue disease, Sjogren’s syndrome, rheumatoid arthritis, and systemic sclerosis. | 134 (26.96) | 298 (59.96) | 65 (13.08) |
| 1. Although no single treatment can completely prevent recurrent miscarriages, maintaining a healthy lifestyle is still a wise choice. | 273 (54.93) | 200 (40.24) | 24 (4.83) |
| 1. If patients with recurrent abortion are diagnosed with antiphospholipid syndrome, heparin, and low-dose aspirin are the basic treatment regimen. | 165 (33.2) | 220 (44.27) | 112 (22.54) |
| 1. If patients with recurrent abortion are diagnosed with hypothyroidism, they need to take levothyroxine. | 123 (24.75) | 187 (37.63) | 187 (37.63) |
| 1. Chromosomal karyotype analysis of both spouses or miscarriage products can help understand the cause of miscarriage; if a chromosomal abnormality is diagnosed, seeking professional genetic counseling is necessary. | 227 (45.67) | 234 (47.08) | 36 (7.24) |
| 1. Currently, there is no evidence to suggest that vitamin D can reduce the risk of miscarriage, but regular intake of vitamin D supplements may reduce the risk of pregnancy complications. | 109 (21.93) | 259 (52.11) | 129 (25.96) |
| 1. Test-tube baby (assisted reproductive technology) is just one technique to assist pregnancy and cannot completely solve the problem of recurrent abortion. | 187 (37.63) | 229 (46.08) | 81 (16.3) |
