# Supplementary material for: Knowledge, attitudes, and practices of patients with recurrent pregnancy loss toward pregnancy loss
Source: Front Public Health. 2024 Jan 11;11:1308842. doi: 10.3389/fpubh.2023.1308842 (PMC10808478; doi:10.3389/fpubh.2023.1308842)
Supplement: Supplementary file 2 [file Table_2.DOCX]

**Supplementary Table S2.** Attitudes of participants

| **Attitude** | | **Strongly agree** | **Agree** | **Neutral** | **Disagree** | **Strongly disagree** |
| --- | --- | --- | --- | --- | --- | --- |
| 1. Biochemical pregnancies have minimal impact on the body and do not affect a woman’s next chance of getting pregnant. (P) | | 57 (11.47) | 187 (37.63) | 107 (21.53) | 104 (20.93) | 42 (8.45) |
| 2. If recurrent abortion is left untreated, the likelihood of it healing on its own is low. (P) | | 133 (26.76) | 295 (59.36) | 53 (10.66) | 12 (2.41) | 4 (0.8) |
| 3. Even for patients with a history of only one miscarriage, a comprehensive etiological examination is necessary. (N) | | 107 (21.53) | 207 (41.65) | 140 (28.17) | 40 (8.05) | 3 (0.6) |
| 4. I believe that miscarriage is a manifestation of undesirable fetal quality and supports the concepts of “natural selection” and “survival of the fittest.” (N) | | 13 (2.62) | 49 (9.86) | 106 (21.33) | 237 (47.69) | 92 (18.51) |
| 5. In the face of the current situation, it is important to identify the cause carefully and try to conceive naturally after treating the cause; it was not necessarily through IVF. (P) | | 132 (26.56) | 278 (55.94) | 74 (14.89) | 11 (2.21) | 2 (0.4) |
| 6. I often feel helpless and isolated when facing the reality of abortion. (N) | | 190 (38.23) | 226 (45.47) | 69 (13.88) | 9 (1.81) | 3 (0.6) |
| 1. I frequently worry about the possibility of recurrent miscarriage, leading to feelings of nervousness and anxiety. (N) | 222 (44.67) | | 215 (43.26) | 49 (9.86) | 8 (1.61) | 3 (0.6) |
| 8. Family care and support are very important to me. (P) | 264 (53.12) | | 210 (42.25) | 21 (4.23) | 1 (0.2) | 1 (0.2) |
| 9. Abortion not only causes me great pain but also imposes significant psychological stress on my partner. (N) | 212 (42.66) | | 230 (46.28) | 52 (10.46) | 3 (0.6) | 0 (0) |
| 10. After experiencing an abortion, I hope to have more positive and open communication with my partner. (P) | 234 (47.08) | | 243 (48.89) | 19 (3.82) | 0 (0) | 1 (0.2) |
| 11. After experiencing an abortion, I do not wish to be treated differently (as if I have a rare disease). (P) | 141 (28.37) | | 226 (45.47) | 88 (17.71) | 35 (7.04) | 7 (1.41) |
| 12. After experiencing an abortion, I hope to receive useful advice from my partner, family, and friends, rather than some layman’s personal opinion or even chastising or changing the subject. (P) | 215 (43.26) | | 236 (47.48) | 38 (7.65) | 6 (1.21) | 2 (0.4) |
| 13. I have complete trust in my attending physician. (P) | 279 (56.14) | | 188 (37.83) | 28 (5.63) | 2 (0.4) | 0 (0) |
