# Supplementary material for: Knowledge, attitudes, and practices of patients with recurrent pregnancy loss toward pregnancy loss
Source: Front Public Health. 2024 Jan 11;11:1308842. doi: 10.3389/fpubh.2023.1308842 (PMC10808478; doi:10.3389/fpubh.2023.1308842)
Supplement: Supplementary file 3 [file Table_3.DOCX]

**Supplementary Table S3.** Practices of the participants

| **Practice** | **Always** | **Often** | **Sometimes** | **Occasionally** | **Never** |
| --- | --- | --- | --- | --- | --- |
| 1. Conduct standardized and personalized etiological screening, treat the underlying causes, and plan for pregnancy after recovery. (P) | 173(34.81) | 202(40.64) | 99(19.92) | 18(3.62) | 5(1.01) |
| 2. If miscarriage is inevitable, opt for a standardized abortion surgery. (P) | 220(44.27) | 107(21.53) | 85(17.1) | 61(12.27) | 24(4.83) |
| 3. Establish good life behaviors: |  |  |  |  |  |
| 3.1 Quit smoking/avoid secondhand smoke (P) | 327(65.79) | 73(14.69) | 41(8.25) | 24(4.83) | 32(6.44) |
| 3.2 Abstain from alcohol (P) | 376(75.65) | 46(9.26) | 21(4.23) | 12(2.41) | 42(8.45) |
| 3.3 Stay in a good mood (P) | 134(26.96) | 195(39.24) | 156(31.39) | 11(2.21) | 1(0.20) |
| 3.4 Engage in moderate exercise, avoiding obesity (P) | 110(22.13) | 145(29.18) | 202(40.64) | 36(7.24) | 4(0.80) |
| 3.5 Avoid prolonged exposure to harmful substances such as heavy metals and pesticides (P) | 267(53.72) | 132(26.56) | 39(7.85) | 39(7.85) | 20(4.02) |
| 3.6 Maintain a balanced diet with attention to balanced nutrition, avoiding raw and cold foods. (P) | 164(33.00) | 203(40.85) | 116(23.34) | 12(2.41) | 2(0.40) |
